# Educational inequalities in all-cause and cause-specific mortality among people with gout: a register-based matched cohort study in southern Sweden

**DOI:** 10.1186/s12939-019-1076-1

**Published:** 2019-10-28

**Authors:** Ali Kiadaliri, Margarita Moreno-Betancur, Aleksandra Turkiewicz, Martin Englund

**Affiliations:** 1 Faculty of Medicine, Department of Clinical Sciences Lund, Orthopaedics, Clinical Epidemiology Unit, Lund University, Lund, Sweden; 2 Centre for Economic Demography, Lund University, Lund, Sweden; 3 Skåne University Hospital, Clinical Epidemiology Unit, Remissgatan 4, SE-221 85 Lund, Sweden; 4 Clinical Epidemiology and Biostatistics Unit, Murdoch Children’s Research Institute, Melbourne, Australia; 5 Centre for Epidemiology and Biostatistics, Melbourne School of Population and Global Health, University of Melbourne, Melbourne, Australia; 6 Clinical Epidemiology Research and Training Unit, Boston University School of Medicine, Boston, MA USA

**Keywords:** Education, Inequality, Gout, Cause-specific mortality, Multiple cause of death, Sweden

## Abstract

**Background:**

Gout is the most common inflammatory arthritis with a rising prevalence around the globe. While educational inequalities in incidence and prevalence of gout have been reported, no previous study investigated educational inequality in mortality among people with gout. The aim of this study was to assess absolute and relative educational inequalities in all-cause and cause-specific mortality among people with gout in comparison with an age- and sex-matched cohort free of gout in southern Sweden.

**Methods:**

We identified all residents aged ≥30 years of Skåne region with doctor-diagnosed gout (ICD-10 code M10, *n* = 24,877) during 1998–2013 and up to 4 randomly selected age- and sex-matched comparators free of gout (reference cohort, *n* = 99,504). These were followed until death, emigration, or end of 2014. We used additive hazards models and Cox regression adjusted for age, sex, marital status, and country of birth to estimate slope and relative indices of inequality (SII/RII). Three cause-of-death attribution approaches were considered for RII estimation: “underlying cause”, “any mention”, and “weighted multiple-cause”.

**Results:**

Gout patients with the lowest education had 1547 (95% CI: 1001, 2092) more deaths per 100,000 person-years compared with those with the highest education. These absolute inequalities were larger than in the reference population (1255, 95% CI: 1038, 1472). While the contribution of cardiovascular (cancer) mortality to these absolute inequalities was greater (smaller) in men with gout than those without, the opposite was seen among women. Relative inequality in all-cause mortality was smaller in gout (RII 1.29 [1.18, 1.41]) than in the reference population (1.46 [1.38, 1.53]). The weighted multiple-cause approach generally led to larger RIIs than the underlying cause approach.

**Conclusions:**

Our register-based matched cohort study showed that low level of education was associated with increased mortality among gout patients. Although the magnitude of relative inequality was smaller in people with gout compared with those without, the absolute inequalities were greater reflecting a major mortality burden among those with lower education.

## Background

Gout is the most common inflammatory arthritis with a rising prevalence around the globe [1]. Previous studies estimated a prevalence range of 0.6%—1.8% and incidence of 16—24 cases per 10,000 person-years for gout in Sweden [2–4]. In addition, Sweden had the 15th highest rate of years lived with disability for gout among 195 countries in 2015 [5]. In addition to causing pain, joint damage, functional impairment, and reduced health-related quality of life, gout is associated with increased mortality [1, 6–8]. A recent study in southern Sweden reported a 17% higher hazard of all-cause mortality among persons with gout than those without gout [8]. Socioeconomic status (SES) including education is a well-documented predictor of health outcomes including mortality. According to the framework proposed by the World Health Organization (WHO) Commission on Social Determinants of Health, SES is considered as a “structural determinant” of health inequalities that through a set of “intermediary determinants” (material circumstances, psychosocial circumstances, behavioural and/or biological factors, and the health system itself) influences exposure and vulnerability to health-compromising conditions [9]. While very few studies assessed associations between SES and incidence and prevalence of gout [4, 10–12], to our knowledge, no previous study investigated association between SES and mortality among people with gout. Assessing educational inequalities in cause-specific mortality provides valuable information to identify major causes responsible for inequalities in mortality. To address this, we assessed the absolute and relative educational inequalities in all-cause and cause-specific mortality among people with gout in comparison with a randomly selected age- and sex matched cohort without gout in southern Sweden.

## Material and method

### Study setting and design

We conducted an observational register-based matched cohort study. The study was conducted in the southernmost region of Sweden, Skåne, with a population of about 1.3 million in 2014 (13.2% of the Sweden’s population).

### Study population

Using the Skåne Health Care Register and the Swedish Population Register, we identified all residents aged ≥30 years who had been diagnosed with gout (the International Classification of Diseases, 10th revision [ICD-10] code M10) by a physician (within primary or secondary care) between 1 January 1998 and 31 December 2013. The date of the first diagnosis of gout was considered as the index date. From the population at risk (i.e. those without a gout diagnosis), we randomly selected up to four comparators free of gout matched by age and sex to each person with gout (the reference cohort). These comparators received the same index date as their gout matched subject. These data were linked with other registers using the personal identification number assigned to all residents in Sweden.

### Level of education and socio-demographic characteristics

We acquired the data on level of education, marital status, and country of birth from the Longitudinal Integration Database for Health Insurance and Labour Market Studies (LISA). We divided the highest level of attained education into three categories: “low” (0–9 years of education), “medium” (10–12 years of education), and “high” (> 12 years of education).

### Outcome and follow-up

The individual-level data on all death certificates issued in the region during 1998–2014 were obtained from the Swedish National Board of Health and Welfare’s Cause of Death Register (http://www.socialstyrelsen.se/). We extracted date of death, the underlying cause of death (UCD), and contributory causes of death according to the ICD-10 from these death certificates. Based on the ICD-10 system, we defined nine main groups of causes of death (infectious diseases, neoplasms, blood & endocrine diseases, mental and nervous diseases, cardiovascular diseases, respiratory diseases, digestive diseases, genitourinary diseases, and other causes). We used the underlying cause of death (UCD) to identify cause-specific deaths in our main analysis, and contributory causes in sensitivity analyses.

Each subject’s follow-up started at the index date or his/her 30th birthday whichever occurred last. All subjects were followed until death, relocation outside Skåne, or end of 2014, whichever occurred first. It should be noted that the exposure ascertainment period was 1998–2013 and the subjects were followed until the end of 2014, to ensure that each included person would have at least one year of observation period.

### Statistical analysis

We assessed relative educational inequalities by estimating the relative index of inequality (RII) – defined as the log-linear association between educational level and mortality, and roughly speaking can be interpreted as the ratio of mortality rates between the two extremes of the educational hierarchy. To estimate RII, each level of education was assigned a fractional rank based on the mean proportion of the population with a higher level of education [13]. Therefore fractional rank is a continuous variable ranging from 0 (the highest education) to 1 (the lowest education). The RII was estimated using cause-specific Cox proportional hazard models [14]. The fractional rank was included as a covariate in these models and the exponential of its coefficient provides an estimate of RII.

We measured the absolute educational inequality by estimating the slope index of inequality (SII) – defined as the linear association between educational level and mortality, and roughly speaking can be interpreted as the absolute difference in mortality rates between the two extremes of the educational hierarchy. The SII was estimated by fitting cause-specific additive hazard model where the coefficient of the fractional rank provides an estimate of SII [13]. The contribution of a specific cause of death to the absolute educational inequality in all-cause mortality was estimated by dividing the SII of that cause by the SII of all-cause mortality.

In all models time since study entry was used as the timescale and all models were adjusted for age (as continuous variable), sex, marital status (never married, previously married, and married), and country of birth (Sweden-born vs. non-Sweden born). Separate models were estimated for those with and without gout. Subgroup analyses by sex and age group (30–74 years, and ≥ 75 years) were also conducted. Analyses were performed using Stata version 15 (data preparation and estimating RIIs for main analysis) and RStudio version 1.1.423 (estimating RIIs for sensitivity analysis using “*survMCOD*” package, and SIIs using “*timereg*” package).

### Sensitivity analysis

In an era characterized by aging population and rising prevalence of multi-morbidity, the UCD might not adequately capture mortality associated with a specific cause [15]. To account for this, we followed two alternative approaches [15] as sensitivity analysis in estimating the cause-specific RIIs: 1) “any mention” approach, where a death with mention of the cause on any part of the death certificate is considered as an event in the Cox models, and 2) “weighted multiple-cause” model, which is an extension of the competing risks Cox model for multiple-cause mortality. This approach requires assigning a positive weight to each cause mentioned on the death certificate such that the sum of the weights per death certificate is equal to one. We considered three weighting strategies: a) equal weights (1/number of causes on a death certificate), b) a weight of 0.5 to the UCD and equal weights to non-underlying causes (0.5/number of non-underlying causes), c) a weight of 0.75 to the UCD and equal weights to non-underlying causes (0.25/number of non-underlying causes). While in the “any mention” approach a death where the certificate mentions more than one cause would be counted as multiple deaths, the “multiple cause of death” approach does not suffer from this limitation. The “any mention” approach has been shown in simulation studies to underestimate standard errors, leading to invalid *p*-values (too low) and confidence intervals (too narrow, with poor coverage probabilities) [15].

## Results

A total of 24,877 patients with a doctor-diagnosed gout during 1998–2013 were identified. We identified 99,504 age- and sex-matched individuals without gout as the reference population (1 patient had no comparator). After exclusion of 842 (3.4%) individuals with missing level of education, we observed 8133 deaths during 127,910 person-years follow up among gout patients (Table 1). In the reference population, we excluded 1670 (1.7%) who did not enter the study (died or emigrated prior to the index date), 3457 (3.5%) with missing data on level of education, and 6 with missing on country of birth. There were 24,051 deaths over 554,054 person-years follow up in the reference population included in the study. In both groups, those with low level of education constituted the largest portion of the sample (45.1% in gout patients vs. 40.2% in the reference population). Cardiovascular diseases (CVDs) accounted for 50.3% of the causes of deaths among people with gout versus 41.2% in the reference population (Supplementary Fig. S1). On the other hand, neoplasms constituted 18.4% of deaths among gout patients versus 24.5% in the reference cohort.

**Table 1:** Baseline characteristics of participants and the number of all-cause and cause-specific deaths, stratified by sex

	Gout patients		The reference population	
	Women	Men	Women	Men
N (%)	7431 (30.9)	16,604 (69.1)	29,258 (31.0)	65,113 (69.0)
Age at entry (years), %				
30–49	6.9	13.9	6.9	13.9
50–64	16.3	26.9	16.7	27.3
65–79	39.1	39.7	39.7	39.9
80+	37.7	19.5	36.7	18.9
Level of education, %				
Low (0–9 years of education)	54.7	40.8	46.9	37.1
Medium (10–12 years of education)	33.6	40.2	35.1	39.2
High (> 12 years of education)	11.7	19.0	18.0	23.7
Marital status at entry, %				
Never married	6.8	12.7	8.8	15.3
Previously married	52.7	23.7	48.2	22.4
Married	40.5	63.5	43.0	62.3
Born in Sweden, %	88.4	88.3	88.4	86.9
Total deaths (%)	2961 (100)	5172 (100)	8525 (100)	15,526 (100)
Infectious diseases (%)	98 (3.3)	133 (2.6)	194 (2.3)	348 (2.2)
Neoplasms (%)	438 (14.8)	1057 (20.4)	1645 (19.3)	4247 (27.4)
Blood & endocrine diseases (%)	146 (4.9)	219 (4.2)	244 (2.9)	459 (3.0)
Mental & nervous diseases (%)	146 (4.9)	191 (3.7)	1043 (12.2)	1166 (7.5)
Cardiovascular diseases (%)	1509 (51.0)	2581 (49.9)	3681 (43.2)	6228 (40.1)
Respiratory diseases (%)	220 (7.4)	324 (6.3)	574 (6.7)	1209 (7.8)
Digestive diseases (%)	106 (3.6)	191 (3.7)	261 (3.1)	469 (3.0)
Genitourinary diseases (%)	92 (3.1)	140 (2.7)	122 (1.4)	257 (1.7)
Other causes (%)	206 (7.0)	336 (6.5)	761 (8.9)	1143 (7.4)
Person-years follow-up	36,488	91,422	165,910	388,117

The RII showed that all-cause mortality rate in the low educated gout patients was 1.3 (95% CI: 1.2, 1.4) times higher than the highly educated ones (Table 2). These educational inequalities persisted across sex and age subgroups. For CVDs as the leading cause of death, the RII was 1.4 (95% CI: 1.2, 1.6). Across remaining causes of death, the RII ranged from 0.89 (95% CI: 0.58, 1.38) for mental and nervous diseases to 1.65 (1.07, 2.55) for blood and endocrine diseases. The magnitude of RII in all-cause mortality among gout patients was smaller than in the reference population (Table 3). The similar pattern was seen across subgroups except women who had almost identical RIIs regardless of their gout status.

**Table 2:** Relative index of inequality (RII) and slope index of inequality (SII) in all-cause and cause-specific mortality among participants with gout, by sex and age

Disease group (ICD-10 codes)	Sexes				Age groups				All (≥30 years)	
	Men		Women		30–74 years		≥75 years			
	RII	SII*	RII	SII*	RII	SII*	RII	SII*	RII	SII*
All-cause	1.29 (1.16, 1.44)	1411 (808, 2014)	1.34 (1.14, 1.57)	2052 (920, 3183)	1.53 (1.28, 1.84)	941 (579, 1304)	1.23 (1.11, 1.36)	2607 (1275, 3939)	1.29 (1.18, 1.41)	1547 (1001, 2092)
Infectious (A00-B99)	0.97 (0.50, 1.88)	−4 (−98, 90)	1.43 (0.58, 3.52)	83 (− 119, 285)	2.24 (0.51, 9.87)	28 (−18, 73)	1.02 (0.59, 1.79)	5 (−221, 233)	1.14 (0.67, 1.95)	23 (− 63, 108)
Neoplasms (C00-D48)	1.15 (0.91, 1.45)	169 (−94, 431)	1.36 (0.91, 2.04)	362 (−98, 823)	1.53 (1.09, 2.15)	289 (86, 493)	1.03 (0.81, 1.32)	68 (− 453, 589)	1.18 (0.97, 1.45)	196 (−36, 429)
Blood & endocrine (D50-D89; E00-E90)	1.27 (0.76, 2.15)	55 (−59, 169)	2.85 (1.23, 6.59)	348 (77, 619)	2.28 (1.02, 5.14)	92 (7, 177)	1.49 (0.90, 2.46)	212 (−49, 472)	1.65 (1.07, 2.55)	132 (26, 238)
Mental & nervous (F00-F99; G00-G99)	1.03 (0.59, 1.80)	3 (− 115, 121)	0.76 (0.38, 1.49)	−108 (− 355, 139)	0.86 (0.32, 2.27)	−8 (−63, 47)	0.92 (0.57, 1.50)	−43 (− 329, 243)	0.89 (0.58, 1.38)	−22 (− 137, 94)
Cardiovascular (I00-I99)	1.44 (1.24, 1.69)	993 (590, 1396)	1.32 (1.05, 1.66)	959 (143, 1776)	1.57 (1.18, 2.08)	407 (160, 654)	1.36 (1.18, 1.56)	1984 (1037, 2931)	1.40 (1.24, 1.60)	990 (611, 1369)
Respiratory (J00-J99)	1.43 (0.91, 2.25)	118 (−30, 265)	1.25 (0.69, 2.27)	119 (− 181, 419)	1.85 (0.85, 4.04)	73 (−7, 154)	1.25 (0.85, 1.84)	193 (− 150, 535)	1.31 (0.92, 1.88)	112 (−19, 243)
Digestive (K00-K93)	1.02 (0.58, 1.77)	7 (−112, 126)	1.28 (0.57, 2.84)	58 (− 146, 262)	1.42 (0.59, 3.44)	35 (−51, 122)	0.91 (0.53, 1.56)	−39 (− 270, 192)	1.04 (0.66, 1.65)	12 (−90, 113)
Genitourinary (N00-N99)	2.11 (1.07, 4.16)	109 (20, 198)	1.05 (0.42, 2.63)	3 (− 207, 213)	4.5 (0.97, 21.25)	54 (2, 106)	1.29 (0.72, 2.28)	99 (−126, 324)	1.56 (0.90, 2.71)	77 (−11, 165)
Other causes	0.92 (0.62, 1.38)	−38 (− 185, 110)	1.64 (0.87, 3.08)	227 (−64, 517)	0.83 (0.42, 1.63)	−29 (−137, 78)	1.18 (0.80, 1.76)	129 (− 188, 446.5)	1.08 (0.78, 1.51)	27 (−109, 163)

Values in parentheses display 95% confidence intervals. All models were adjusted for age, sex, marital status, and country of birth

* Per 100,000 person-year

**Table 3:** Relative index of inequality (RII) and slope index of inequality (SII) in all-cause and cause-specific mortality among participants without gout, by sex and age

Disease group (ICD-10 codes)	Sexes				Age groups				All (≥30 years)	
	Men		Women		30–74 years		≥75 years			
	RII	SII*	RII	SII*	RII	SII*	RII	SII*	RII	SII*
All-cause	1.51 (1.42, 1.61)	1320 (1079, 1560)	1.33 (1.21, 1.45)	1139 (734, 1543)	1.78 (1.62, 1.97)	952 (812, 1092)	1.33 (1.25, 1.41)	2587 (2002, 3172)	1.46 (1.38, 1.53)	1255 (1038, 1472)
Infectious (A00-B99)	1.31 (0.87, 1.96)	13 (−22, 48)	1.52 (0.81, 2.84)	34 (−25, 93)	2.44 (1.20, 4.98)	24 (5, 43)	1.18 (0.82, 1.72)	30 (−60, 121)	1.38 (0.99, 1.93)	19 (−11, 49)
Neoplasms (C00-D48)	1.34 (1.19, 1.51)	276 (153, 398)	1.06 (0.87, 1.29)	63 (−132, 258)	1.43 (1.23, 1.67)	237 (148, 327)	1.08 (0.95, 1.23)	144 (− 116, 404)	1.24 (1.12, 1.37)	182 (76, 287)
Blood & endocrine (D50-D89; E00-E90)	1.11 (0.78, 1.57)	3 (−39, 44)	2.03 (1.16, 3.57)	90 (21, 159)	1.59 (0.94, 2.69)	24 (0, 48)	1.25 (0.88, 1.76)	57 (−40, 154)	1.34 (1.00, 1.79)	27 (−10, 63)
Mental & nervous (F00-F99; G00-G99)	1.16 (0.92, 1.45)	7 (−54, 68)	1.04 (0.80, 1.34)	−11 (− 155, 132)	1.23 (0.82, 1.84)	25 (−11, 60)	1.04 (0.87, 1.25)	18 (−160, 195)	1.10 (0.93, 1.30)	14 (−51, 78)
Cardiovascular (I00-I99)	1.67 (1.51, 1.84)	652 (499, 805)	1.57 (1.36, 1.81)	733 (464, 1002)	2.19 (1.84, 2.61)	409 (325, 494)	1.49 (1.36, 1.63)	1586 (1195, 1977)	1.65 (1.52, 1.79)	678 (544, 812)
Respiratory (J00-J99)	2.27 (1.80, 2.87)	209 (143, 276)	1.43 (1.00, 2.05)	102 (−4, 208)	2.69 (1.81, 3.99)	101 (65, 137)	1.76 (1.42, 2.19)	412 (257, 567)	2.01 (1.65, 2.43)	174 (120, 229)
Digestive (K00-K93)	1.51 (1.05, 2.16)	43 (0, 85)	1.82 (1.06, 3.12)	81 (5, 157)	2.54 (1.49, 4.33)	50 (22, 79)	1.37 (0.97, 1.94)	86 (−15, 186)	1.65 (1.23, 2.22)	56 (20, 93)
Genitourinary (N00-N99)	1.49 (0.93, 2.40)	17 (−14, 47)	1.59 (0.75, 3.40)	20 (−27, 66)	1.77 (0.63, 4.96)	9 (−7, 24)	1.31 (0.85, 2.02)	42 (−34, 118)	1.45 (0.97, 2.17)	14 (−9, 38)
Other causes	1.54 (1.22, 1.93)	101 (35, 167)	1.19 (0.88, 1.62)	27 (−91, 145)	1.87 (1.30, 2.69)	72 (30, 115)	1.39 (1.12, 1.71)	213 (57, 369)	1.45 (1.21, 1.74)	92 (35, 149)

Values in parentheses display 95% confidence intervals. All models were adjusted for age, sex, marital status, and country of birth

* Per 100,000 person-year

**Fig. 1:**
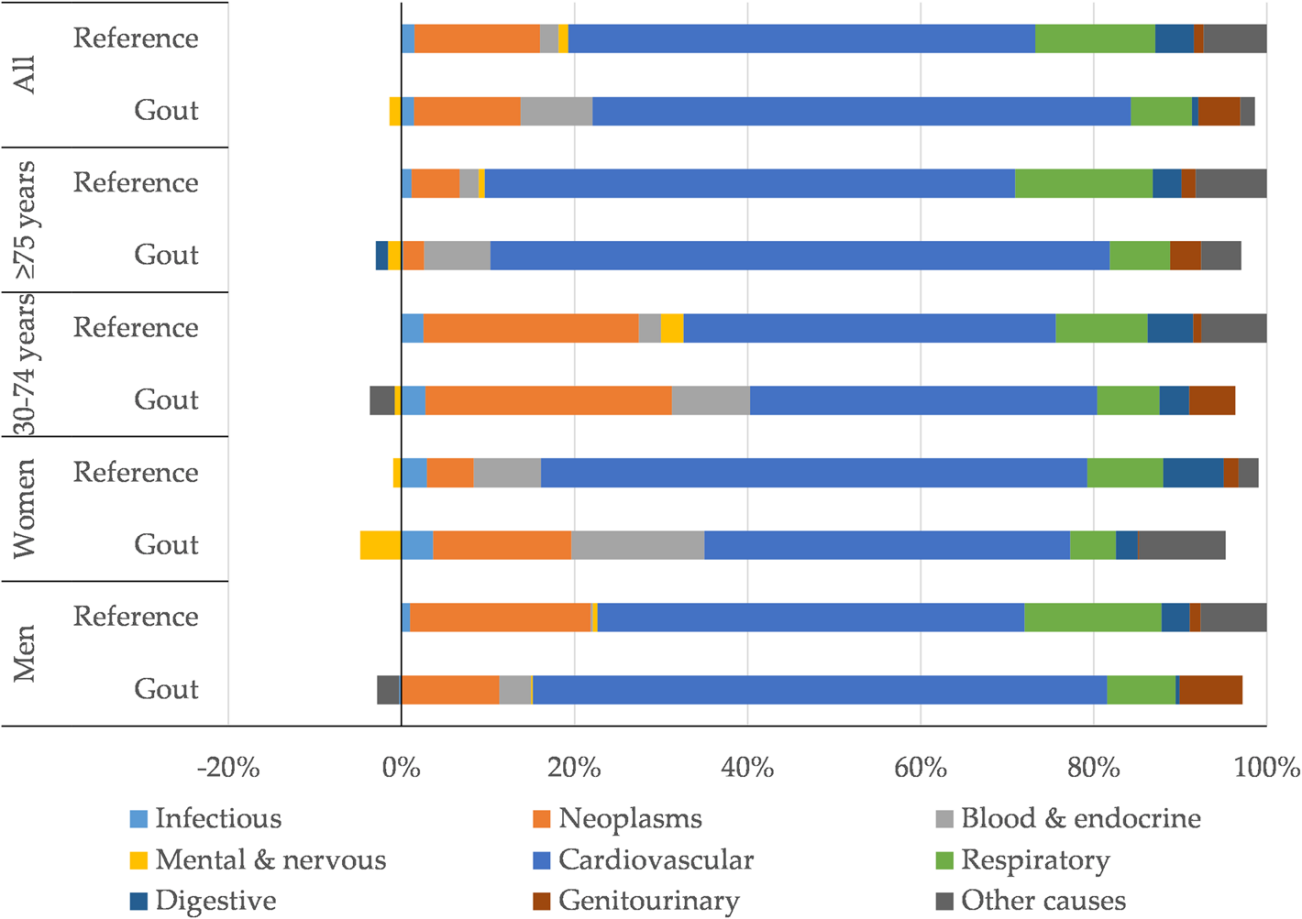
Contribution of specific causes of death to the absolute inequality in all-cause mortality

The SII revealed that there were, on average, 1547 (95% CI: 1001, 2092) more deaths per 100,000 person-years in the least vs. most educated gout patients. CVDs made the largest contribution (64%) to this difference, followed by neoplasms (12.7%) and blood& endocrine diseases (8.5%, Fig. 1). The magnitude of absolute educational inequality in all-cause mortality was greater in people with gout compared with those without (SII 1255 per 100,000 person-years, 95% CI: 1038, 1472). The contributions of CVDs (64% vs. 54%), blood & endocrine diseases (8.5% vs. 2.2%), and genitourinary diseases (5.0% vs. 1.1%) to the absolute inequalities in all-cause mortality were more pronounced in gout patients than in the reference population. While among people with gout, the absolute educational inequality in all-cause mortality was greater in women than men (2052 vs. 1411 deaths per 100,000 person-years), the opposite was observed among those without gout (1139 vs. 1320 deaths per 100,000 person-years). In both groups, the SIIs were larger in people aged≥75 years than younger ones. There were variations by age, sex, cause, and gout status in the patterns of absolute educational inequalities and contributions of causes of death to these inequalities. For instance, in both groups, neoplasms had a substantially larger contribution to the absolute educational inequalities in all-cause mortality among younger people compared with those older.

The magnitude of relative inequalities were generally greater using the “weighted multiple-cause” approach compared with the UCD approach (further distance from 1). Furthermore, in contrast with the UCD approach, the “weighted multiple-cause” approach did not evidence an inverse association between education and cause-specific mortality for infectious diseases, digestive diseases, and other causes among gout patients. As expected, the “any mention” approach resulted in narrower 95% CIs for RIIs (Table 4).

**Table 4:** Relative index of inequality (95% confidence interval) using different cause-of-death attribution approaches

	Participants with gout					Participants without gout				
	Underlying cause	Any mention	Multiple cause			Underlying cause	Any mention	Multiple cause		
Disease group	ω = 1		ω = 0.75	ω = 0.5	Equal weights	ω = 1		ω = 0.75	ω = 0.5	Equal weights
Infectious	1.14 (0.67, 1.95)	1.36 (1.00, 1.86)	0.76 (0.15, 3.93)	0.76 (0.15, 3.99)	0.76 (0.15, 3.97)	1.38 (0.99, 1.93)	1.40 (1.15, 1.70)	1.63 (0.63, 4.20)	2.61 (0.83, 1.95)	2.46 (0.79, 7.67)
Neoplasms	1.18 (0.97, 1.45)	1.21 (1.01, 1.44)	1.11 (0.87, 1.42)	1.06 (0.8, 1.41)	1.04 (0.77, 1.40)	1.24 (1.12, 1.37)	1.26 (1.15, 1.38)	1.20 (1.07, 1.35)	1.19 (1.05, 1.36)	1.17 (1.02, 1.34)
Blood & endocrine	1.65 (1.07, 2.55)	1.48 (1.21, 1.81)	5.81 (2.30, 14.65)	2.27 (0.17, 30.32)	2.26 (0.18, 27.80)	1.34 (1.00, 1.79)	1.71 (1.48, 1.96)	NA	1.56 (0.19, 13.06)	1.58 (0.19, 13.25)
Mental & nervous	0.89 (0.58, 1.38)	1.00 (0.79, 1.27)	0.58 (0.23, 1.41)	0.48 (0.19, 1.18)	0.52 (0.21, 1.27)	1.10 (0.93, 1.30)	1.25 (1.12, 1.39)	0.94 (0.71, 1.25)	0.86 (0.63, 1.17)	1.10 (0.74, 1.63)
Cardiovascular	1.40 (1.24, 1.60)	1.39 (1.26, 1.55)	1.44 (1.26, 1.65)	1.46 (1.27, 1.68)	1.50 (1.27, 1.76)	1.65 (1.52, 1.79)	1.60 (1.50, 1.71)	1.72 (1.57, 1.88)	1.75 (1.59, 1.93)	1.82 (1.64, 2.01)
Respiratory	1.31 (0.92, 1.88)	1.47 (1.22, 1.77)	1.51 (0.59, 3.85)	1.74 (0.68, 4.43)	1.75 (0.75, 4.08)	2.01 (1.65, 2.43)	1.70 (1.53, 1.89)	2.25 (1.52, 3.32)	2.12 (1.55, 2.90)	1.60 (1.06, 2.42)
Digestive	1.04 (0.66, 1.65)	0.98 (0.73, 1.32)	1.04 (0.33, 3.25)	0.81 (0.24, 2.68)	0.87 (0.31, 2.46)	1.65 (1.23, 2.22)	1.64 (1.36, 1.98)	2.13 (1.00, 4.52)	2.12 (1.00, 4.50)	2.12 (1.00, 4.50)
Genitourinary	1.56 (0.90, 2.71)	1.45 (1.18, 1.77)	1.80 (1.03, 3.15)	3.14 (0.63, 15.72)	3.12 (0.63, 15.52)	1.45 (0.97, 2.17)	1.47 (1.25, 1.72)	1.17 (0.50, 2.75)	NA	NA
Other	1.08 (0.78, 1.51)	1.20 (1.04, 1.38)	0.91 (0.56, 1.49)	0.84 (0.55, 1.29)	0.84 (0.53, 1.32)	1.45 (1.21, 1.74)	1.48 (1.36, 1.61)	1.41 (1.11, 1.78)	1.59 (1.31, 1.93)	1.57 (1.29, 1.90)

ω: weight attributed to the underlying cause of death; NA: these models encounter convergence problem. All models were adjusted for age, sex, marital status, and country of birth

## Discussion

In this study, for the first time, we quantified the absolute and relative educational inequalities in all-cause and cause-specific mortality in gout patients and contrasted these with the estimates from a randomly selected age- and sex matched cohort free of gout. Our results suggested that while the magnitude of relative educational inequalities were smaller in gout patients compared with the reference population, the former experienced greater degree of the absolute educational inequalities reflecting a larger mortality burden. Although CVDs were the main driver of absolute educational inequalities in both groups, their relative importance was more pronounced in gout patients than in the reference population. The strengths of associations between level of education and mortality varied by cause of death and its attribution approach, sex, as well as age.

Previous studies have relatively consistently shown that people with lower SES have higher incidence and prevalence of gout [4, 10, 11], suffer from more severe gout [16], and are more prone to discontinue therapy [17]. In our sample, gout patients were, on average, less educated than their non-gout comparators. In addition, higher prevalence of unhealthy behaviours (e.g. alcohol consumption, smoking, physical inactivity) and multi-morbidity among low educated people might partially explain the observed inequalities in our study [18, 19]. Furthermore, despite universal healthcare access in Sweden, educational differences in healthcare utilisation should not be overlooked.

Our results revealed that the magnitude of relative educational inequalities were less pronounced in people with gout than those without. While underlying pathways for this finding need further investigation, one potential explanation is that contacts with healthcare service might decline educational inequalities in health and mortality risk among patients with gout. In contrast, the magnitude of absolute educational inequality in all-cause mortality was larger in gout patients than in the reference population. This finding is mainly driven by larger absolute inequalities in deaths from CVDs and blood & endocrine diseases in gout patients compared with the reference population. Smaller relative inequalities and larger absolute inequalities in a disease group compared with those without the disease has previously been reported [20, 21]. This finding highlights the importance of assessing both relative and absolute inequalities in presenting an accurate picture of socioeconomic inequalities in health.

The substantial contribution of CVDs to the absolute inequalities in all-cause mortality highlights the need for improved prevention and treatment of these diseases among gout patients with lower education, particularly considering that gout patients have a higher CVDs mortality rate than those without gout [6]. The contribution of CVDs to the absolute inequalities in all-cause mortality rose with age while the opposite was observed for neoplasms. Similar patterns have been observed in the reference population in our study and also in the general population in other countries and have been attributed to reduction in socioeconomic differences in the prevalence of risk factors for cancer (e.g. smoking) with increasing age [22].

Quantifying, for the first time, both absolute and relative educational inequalities in all-cause and several cause-specific mortality in a large cohort of gout patients and comparing it with those without gout are the main strengths of the current study. Furthermore, we applied the recent methodology advances in studying SII [13] and multiple-cause of death data [15]. Despite these, several limitations of the current study should be acknowledged. We studied only gout patients diagnosed by a physician and possibility of misdiagnosis and occasional coding errors cannot be ruled out, but is expected to be non-differential. While Sweden has a high quality cause of death register [23], death certificates are subject to misclassification including over-reporting of some causes (e.g. CVDs). If there are educational differences in quality of cause of death data, then our estimates would be biased. However, a previous study found no educational differences in the use of ill-defined causes of death in Sweden [24]. Due to the lack of data, we did not control for several important confounders of the education-mortality or (age at diagnosis of) gout-mortality associations, which could lead to confounding or selection bias, respectively. These include, for instance, cognitive ability, family background, body mass index, and early life health status. This suggests that no causal inference should be made from the findings. It also should be noted that the reference cohort in our study included age-and sex-matched comparators free of gout which might not be representative of the general population and hence any extrapolation of the results to the general population should be avoided.

## Conclusion

Our study showed that low level of education was associated with increased mortality among gout patients. Although the magnitude of relative inequality was smaller in people with gout than in the reference cohort, the absolute inequality was larger in those with gout reflecting a major mortality burden among those with lower education, especially low educated women. The strength of these associations varied by cause of death and its attribution approach, sex, and age. Our results call for improvements in management of gout and comorbidities in Sweden. In particular, decreasing exposure to CVDs risk factors through implementing effective interventions for all, with greater intensity for low educated individuals, should be a public health priority.

## Supplementary information

**Additional file 1: Fig. S1.** The distribution of causes of death in the whole sample and across education groups.

## Data Availability

No additional data is available.

## Abbreviations

CI: Confidence interval
CVDs: Cardiovascular diseases
RII: Relative index of inequality
SES: Socioeconomic status
SII: Slope index of inequality
UCD: Underlying cause of death
