# Supplementary figures and images for: Educational inequalities in all-cause and cause-specific mortality among people with gout: a register-based matched cohort study in southern Sweden
Source: Int J Equity Health. 2019 Oct 28;18:164. doi: 10.1186/s12939-019-1076-1 (PMC6819587; doi:10.1186/s12939-019-1076-1)

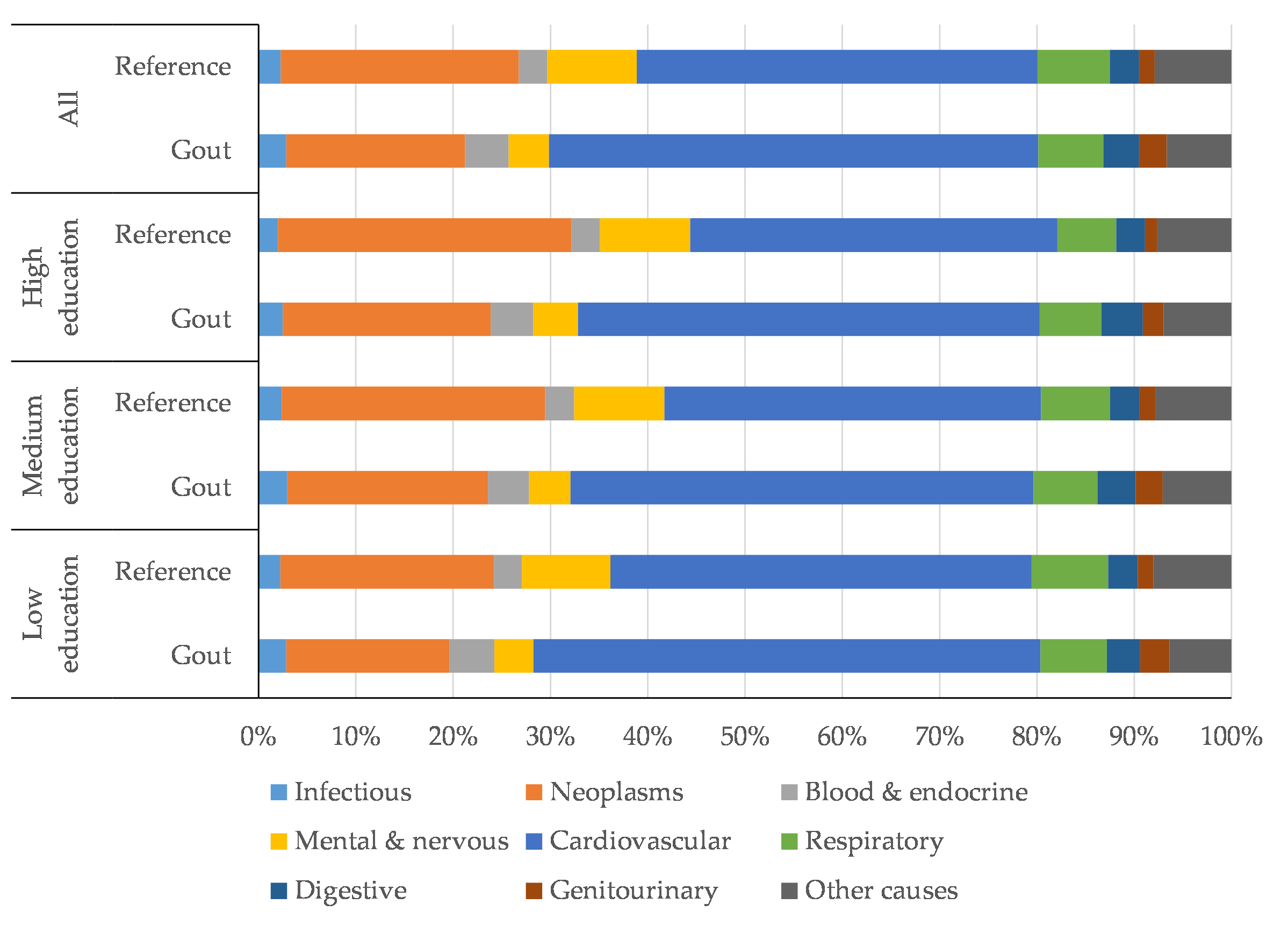

Supplement: Supplementary file 1 — Additional file 1: Fig. S1. The distribution of causes of death in the whole sample and across education groups. [file 12939_2019_1076_MOESM1_ESM.tiff]
